# Reviewing the Effect of English as a Foreign Language Teachers’ Positive and Negative Affectivity on Their Work Engagement

**DOI:** 10.3389/fpsyg.2022.852687

**Published:** 2022-02-28

**Authors:** Yuguo Fan

**Affiliations:** School of Foreign Languages, Qilu Normal University, Jinan, China

**Keywords:** boredom, enjoyment, frustration, negative emotions, positive emotions, pride, shame, work engagement

## Abstract

This review strives to illuminate the related studies on the effect of English as a Foreign Language (EFL) teachers’ positive and negative emotions on their work engagement. The negative correlations among teachers’ boredom, apprehension, shame, frustration, and work engagement have been confirmed in the review of the literature. Furthermore, few studies have validated the effect of teachers’ positive emotions, such as enjoyment and pride, on their work engagement in educational contexts. The studies showed that some factors, such as teacher self-efficacy, teacher self-sufficiency, increased academic challenges, and ambiguity in educational contexts, can mediate the relationship between teachers’ negative emotions and work engagement. The review of literature has emphasized the mediating role of growth mindset in the relationship between teachers’ positive emotions and work engagement. To improve the language teaching quality, the pedagogical implications are explained in the end. Some suggestions for further research are provided to expand the literature about teachers’ emotional variables.

## Introduction

Recently, emotions have been regarded as important factors influencing teachers’ behavior in instructive contexts. In effect, many studies have tended to explore teacher emotions and their effects on teacher-student interpersonal relationships, learners’ academic achievement, and language proficiency (Xie and Derakhshan, 2021). Language teachers are required not only to have language proficiency, the capability of designing approaches and methods, the ability to assess and evaluate learners, and the aptitude to use numerous instruction aids, but they also should build their identity through considering their negative and positive affectivities (Liu, 2016). It is required to view various features which can negatively and positively affect teacher quality in L2 contexts (Fathi and Derakhshan, 2019). Traditionally, psychologists have highlighted the deficiencies and negative emotions among learners and teachers, and they have made an effort to decrease them (Deweale and Alfawzan, 2018; Derakhshan et al., 2021). However, the investigation of negative construct effects on learners’ academic enjoyment, engagement, and performance was insufficient. Therefore, positive psychology (PP) has recently emerged, and many positive psychologists have verified that the consideration of learners’ strength to improve their learning outcome has been effective for many investigators (MacIntyre et al., 2019; Wang et al., 2021). It tries to illuminate the optimal educational situations and processes for the achievement of learners and teachers (Jiang, 2020). However, investigators tended to investigate the constructs of positive emotions in more detail with the hope of assisting language learners and teachers to process language better in their minds (Fang and Tang, 2021).

Exploration of this review will be significant in language learning education. It has actually been suggested that the awareness of positive and negative emotions helps teachers regulate their emotions to have successful instruction. This review gives a significant advantage because language instructors can obtain a complete understanding of their negative and positive emotions.

The findings of this study may help to encourage teachers to be more aware of their perceptions since, according to humans’ perceptions affect their actions and emotions. This review seeks to investigate the effect of positive affectivity as one of the constructs of positive psychology and negative affectivity of teachers on their work engagement.

## Literature Review

### Emotion

According to Frenzel and Stephens (2013, p. 5), emotion refers to “multidimensional constructs comprising affective, psychological, cognitive, expressive, and motivational components”. Lauermann and Butler (2021) pointed out that the concept of emotion signifies individuals’ physiological and sensual feelings relating to their mental and physical conditions. Since teachers’ emotions, in the educational contexts, are related to their behavior, teacher-learner relationships, and learner performance, the investigation of teachers’ emotional factors is critical to realize the instructional approaches (Urhahne, 2015). In the learning contexts, instructors may be subjected to various positive and negative feelings as a result of interaction with their learners, coworkers, mentors, and principals (Chang, 2009). Balanescu (2019) argued that teacher performance, used methodology, and learners’ language proficiency are affected by their teachers’ positive and negative affections.

### Positive Emotions

With the advent of positive psychology in foreign language studies, most investigators tended to study the facilitative influence of positive affectivity or emotion in foreign language contexts (White, 2018). Pekrun (2014) argued that positive affectivity promotes learning by expanding individuals’ attention to the numerous learning tasks and increasing their motivation. Pekrun and Perry (2014) stated that proficient learners’ use of learning strategies is correlated with their positive affectivity. Moreover, Schunk and Jeffrey (2018) argued that reflecting and planning methods, as two types of learning self-regulation, are positively associated with pleasant emotions, and their interactions may result in achievement in language learning. Moreover, Figueira et al. (2018) study revealed that individuals’ working memory is positively correlated with their pleasant emotions since positive emotions can expand individuals’ capability to handle information. Bastian et al. (2014) also showed that individuals’ positive affectivity can increase their contentment and subjective wellbeing. Nalipay et al. (2019) argued that teachers, with higher positive affectivity, tend to use and generate novel teaching approaches. They mentioned that teachers should also develop favorable educational contexts to increase positive emotions among learners. Liu (2016) also mentioned that teachers who steadily control their positive emotions and use their chances to gain new knowledge can develop their profession. Frenzel (2014) categorized positive emotions into enjoyment and pride.

Mierzwa (2019) pointed out that enjoyment is the result of learners’ perceptions over their competence and performance in instructive tasks. Numerous studies have been done on the role of learners’ enjoyment in educational contexts. Piniel and Albert (2018) argued that enjoyment and anxiety were the key points within the emotion network. Hagenauer and Hascher (2014) categorized the construct of enjoyment into behavioral, mental, emotional, expressional, and psychological facets. They mentioned that the affective aspect, like enjoyment, relates to the learners’ emotions and satisfaction, and the mental dimension is associated with learners’ positive attitude. Han and Wang (2021) also argued that enjoyment is the result of enthusiasm. Recently some investigations have been done on the enjoyment construct in the EFL contexts. Dewaele and MacIntyre (2014), in their study, investigated EFL learners’ enjoyment and their level of classroom apprehension. They found out that EL learners’ level of enjoyment is higher than their anxiety. They argued that learners’ level of foreign language enjoyment is related to their education and foreign language proficiency. Deweale and Alfawzan (2018) also found out that enjoyment is significantly correlated with the overall scores of learners in foreign language skills. Lately, Elahi Shirvan et al. (2021) studied learners’ foreign language enjoyment at different time points, and their results indicated the time-based fluctuation of foreign language enjoyment. Botes et al. (2021) study revealed a significant positive correlation between enjoyment, engagement, motivational intensity, and learners’ willingness to communicate. Méndez-Aguado et al. (2020) investigated the effect of enjoyment on motivation, and they found out that enjoyment is significantly correlated with motivation. Sampson (2020), in his study, found out that learners’ enjoyment is significantly affected by EFL teachers’ behaviors and friendliness, peer cooperation, learners’ language proficiency, and their attitudes toward their instructors. Jiang (2020) also found that instructors’ traits, pleasure, wit, sociability, compassion, and endurance can significantly predict learners’ foreign language enjoyment. Jiang and Dewaele (2019) highlighted that EFL learners’ enjoyment is significantly associated with the teacher’s behavioral variables. They found out that (Deweale and Alfawzan, 2018)instructor’s outgoingness was strongly correlated with learners’ enjoyment. Li et al. (2021) mentioned that teachers can cause enjoyment and emotional support among Chinese EFL learners by providing a positive classroom context. Burić and Moè (2020) stated that good instruction is full of positive emotions. Keller et al. (2014) considered enjoyment one of the most tangible feelings that instructors experience in educational contexts. Talebzadeh et al. (2020) found the transmission of enjoyment from instructor to learners. Their study revealed that enjoyment was conveyed by the instructor to the learner through facial expressions, posture, movement, and vocalization.

Frenzel (2014) also mentioned that pride can be considered as a dominant feeling following enjoyment. Keller et al. (2014) also mentioned that instructors’ achievements and learners’ successes affect the instructors’ pride. Toraby and Modarresi (2018), in their study, found out that teachers’ enjoyment and pride are significant predictors of learners’ achievement. Dewaele (2015) recognized that using a foreign language and interest in the educational contexts can increase teachers’ enjoyment and pride. Goetz et al. (2007) studied the relationship between positive affectivity factors, including enjoyment and pride, and negative ones, including anger, and boredom in various domains, such as English, German, mathematics, and physics classrooms. Their study revealed the context-based differences in the relationships between positive and negative affectivity. They also found strong associations between positive and negative emotions in similar subject domains.

### Negative Emotions

Frenzel (2014) classified negative affectivities into boredom, shame, apprehension, and frustration. Macklem (2015) pointed out that boredom is a concept that relates to the feeling of purposeless and indifference. He maintained that boredom is described as a negative emotional experience, taking place in individuals’ environment when it is supposed as uninteresting and wearisome. Fahlman et al. (2013) argued that teachers, who feel bored, are usually dissatisfied with their jobs, and they have less inspiration to follow their objectives. Pawlak et al. (2020) classified the construct of boredom into a trait (permanent) and state (impermanent) aspects. They maintained that task enjoyment and the learning process are negatively affected by the instructors’ boredom. Nakamura et al. (2021) argued that there are some reasons for boredom, including the lack of meeting teachers and learners’ interests and expectations, learners’ feelings about their incompetency, the existence of over-challenging, and under-challenging tasks, and input flooding without any new ideas. Derakhshan et al. (2021) also argued that instructors’ lengthy, uninteresting monologs, academic disengagement, logistical difficulties, and inaccurately selected, tedious tasks were the key causes of boredom. Taxer and Frenzel’s (2015) study revealed the negative correlations between teachers’ boredom and their wellbeing, self-efficacy, and rapport. Moreover, they found boredom as the strongest predictor of teachers’ job dissatisfaction. Khajavy et al. (2018) investigated the reasons for negative and positive affections among language teachers. They found discouraged and uncollaborative learners as the reasons for teacher boredom. Dumančić (2018) found the negative influence of teacher boredom on teaching quality. He argued that a supportive environment in EFL contexts encourages teaching quality. Tam et al. (2020) investigated the effect of teacher boredom on learners’ boredom and language proficiency. They found that teacher boredom is strongly correlated with learners’ insights over teacher boredom and with learners’ language proficiency. Therefore, they argued that teacher boredom enhances learners’ own sense of boredom, which results in a reduction in language learning proficiency.

Frenzel (2014, p. 7) called shame as another aspect of negative affection in the educational contexts, and he defined it as a “global negative evaluation of the self in an uncontrollable situation”. Bibby (2002) argued that shame depends on cultural context, and many eastern cultures, unlike western countries, are dealing with shame in their culture. He argued that shame can be induced by outside negative judgments. Frenzel (2014) stated that teachers, in the instructional environments, are probably assessed negatively by the learners. This leads to shame as an emotional state. However, he argued that shame is not a noticeable emotion for instructors. He attributed his result to the approach in the assessment of shame. Karimi and Fallah (2021) found out that teachers’ positive emotions, shame, and motivation are significantly correlated with learners’ academic burnout. Their study also revealed that teachers’ positive emotions and supports are indirectly related to learners’ academic burnout *via* teachers’ motivation and shame as mediators.

Numerous studies have been done on EFL learners’ apprehension in language learning contexts (e.g., Guo et al., 2018; Lubis, 2020; Aslan and Thompson, 2021; York et al., 2021). Given that teacher apprehension is regarded as one of the negative affections in educational contexts (Frenzel, 2014), few investigations have been done on this issue in EFL contexts (e.g., Kralova and Tirpakova, 2019; Ghanizadeh et al., 2020; Goldast et al., 2021). Kyriacou (2001) defined apprehension as the future-based anxiety and stress which occur in unpleasant specialized contexts. He mentioned that anxious individuals usually have some problems communicating with people. In the teaching context, internal and external stressors are experienced by teachers. Being incompetent in communication, feeling lonely, and lacking self-esteem are examples of internal stressors. On the other hand, having insufficient knowledge about recent instructional materials, inefficient rapport with learners, time pressure during instruction, and struggling with demotivated learners can be the instances of external stressors (Kyriacou, 2001). Communication apprehension is a common type of teacher apprehension that is highly related to nervousness about interacting with learners (Kim and Kim, 2004). Since communication apprehension is a fundamental issue that restrains the received comprehensible input, it plays a key role in specifying achievement in language learning and teaching (Darmawangsa et al., 2020). Goldast et al. (2021) indicated some teachers’ lack of self-esteem, L2-related problems, cultural differences, classroom management, and organizational problems are significantly related to teachers’ apprehension. In a study, Ghanizadeh et al. (2020) found that instructors’ identity and self-esteem are significantly correlated with teachers’ apprehension. Shillingford-Butle et al. (2012) also argued that teacher-parent interactions, instructive policies, and struggling with other instructors can lead to teacher apprehension. Kralova and Tirpakova (2019) found a positive relationship between teacher apprehension and age. They also found that non-native teachers are more anxious than native ones since they assume that their language proficiency is inadequate.

Frustration, as another aspect of negative emotion (Frenzel, 2014), refers to an undesirable feeling when learners are not allowed to reach their objectives or change unwanted circumstances (Kuppens et al., 2008). Frustration is regarded as an expediter of teacher burnout which is defined as “a syndrome of emotional exhaustion, lack of personal accomplishments, and depersonalization and the fire of enthusiasm and commitment to success being reduced to ashes” (Maslach, 1976, p. 1). Lazarus (1991) argued that frustration leads to irritation, apprehension, and unhappiness. He also maintained that frustration is regarded as a sign of anger. King (2016) also stated that frustration is caused by student silence, overall misbehavior, and organizational maladministration. Morris and King (2018) argued that language instructors can regulate their emotions and lower their frustration through realizing learners’ socio-cultural backgrounds. They mentioned that teachers should use emotional regulation strategies in order to lessen their frustration.

### Work Engagement

Schaufeli et al. (2002, p. 75) pointed out that work engagement refers to “a positive, fulfilling, work-related state of mind that is characterized by vigor, dedication, and absorption”. Hakanen et al. (2006, p. 498) described vigor as “high levels of energy and mental resilience while working, the willingness to invest effort in one’s work, and persistence also in the face of difficulties”. They mentioned that the feelings of eagerness, motivation, arrogance, and challenges are related to dedication. They also maintained that absorption, as another aspect of work engagement, is related to the full concentration of work.

#### The Effect of Positive Emotions on Work Engagement

Most studies have been done on the influence of learners’ and instructors’ positive emotions and their relations with learners’ engagement in foreign language contexts (Derakhshan, 2021; Liu, 2021; Xie and Derakhshan, 2021). Some studies have verified the relationship between work engagement of teachers and variables, such as social support and self-efficacy (Minghui et al., 2018), teacher identity (Van Der Want et al., 2019), teachers’ psychological wellbeing, and emotion regulation (Greenier et al., 2021), and learners’ academic engagement (Zhang and Yang, 2021). Few studies have been done on the relationship between teachers’ positive emotions and work engagement. Zeng et al. (2019) found out that teachers with the growth mindset experience enjoyable challenges, and they found out that wellbeing and incremental mindset act as mediator variables in the correlation between enjoyment and work engagement. Frondozo et al. (2020) also stated that teachers’ growth mindset about their instructional capability is significantly correlated with their enjoyment and work engagement. They argued that instructors with a growth mindset in the instructional process usually regard teaching as a flexible job, and they assume activities as chances to improve their capabilities, and this results in enjoyment and work engagement. Jin and Zhang (2019) declared that enjoyment can cause engagement in learning contexts and increasing social-behavioral learning engagement. They underscored that enjoyment result in continual determination along with engagement in instructive situations. Nalipay et al. (2020) highlighted the role of enjoyment and emotional enablement in developing instructors’ work engagement. Guo (2021) study revealed that teachers’ higher enjoyment and lower anger mediate the correlation between teachers’ self-efficacy and work engagement.

### The Effect of Negative Emotions on Work Engagement

Some investigations have been done on the relationship between teachers’ negative emotions and work engagement in the educational contexts. Burić and Macuka (2018) investigated the mutual relationship between teachers’ negative emotions and work engagement. They found a negative correlation between negative emotions and work engagement. They argued that frustration, desperateness, and weariness cause less engagement among teachers. Moreover, they found out that teacher self-efficacy plays a mediator role in the relationship between negative emotions and work engagement. Skaalvik and Skaalvik (2014) stated that teacher self-sufficiency and self-efficacy mediate the relationship between work engagement and teacher fatigue. Slišković et al. (2019) mentioned that frustration and hopelessness among teachers are regarded as interrupting reasons for work engagement. Macklem (2015) stated that teacher boredom negatively influences work engagement, and he recommended overcoming teacher boredom to stop teacher disengagement. He maintained that the active participation of teachers needs to be reinforced in L2 contexts to avoid troublesome conduct and decrease the valence of negative feelings, including anxiety, frustration, and boredom. Eren (2016), in his study, found out that teachers’ boredom is negatively correlated with their cognitive, emotional, and work engagement. Harju et al. (2016) study implicated that the expansion of challenges in educational contexts is negatively correlated with teacher boredom and positively correlated with work engagement. Mérida-López et al. (2017) found a predictive role of teacher anxiety in work engagement. They argued that vagueness in educational contexts and stress result in emotional distress and decreased job engagement. Van Der Want et al. (2019) found a negatively significant relationship between teacher burnout and work engagement. Hakanen et al. (2006) found out that job resources, including job management, social, and supervisory support, are negatively correlated with burnout, and burnout, in turn, is negatively correlated with teacher engagement. Kulavuz-Onal and Tatar (2017) argued that quality enhancement of educational contexts along with the provision of motivations for teachers to participate in language tasks would probably boost teachers’ professional development, which results in reduced teacher burnout and frustration, and increased work engagement. Mojsa-Kaja et al.’s (2015) study revealed that teachers who suffer from burnout, compared to involved ones, cannot adapt themselves to the working environment. They argued that teachers with higher levels of work engagement have lower levels of negative emotions.

## Implications and Suggestions

Teachers’ negative and positive feelings are critical for the growth of instruction in educational contexts. This review examined the effect of teachers’ positive and negative emotions on their work engagement. To do so, Frenzel’s (2014) model was used in this review. This review improved our knowledge about teachers’ negative emotions, including apprehension, boredom, shame, frustration, and positive emotions, such as enjoyment and pride, and their effects on teachers’ work engagement. The review of literature verified the significant and negative effects of boredom, anxiety, frustration, and shame on teachers’ work engagement. Moreover, it has been proved that teachers’ positive emotions, including enjoyment and pride, have a significant effect on their work engagement. This review enhances the educational knowledge of investigators who are interested in teachers’ emotions. Considering the related studies on the role of learners’ affective factors on teacher engagement, it can be mentioned that learners should be assisted to control, adjust, and regulate their emotions in language learning contexts to help teachers engage in classroom contexts. Language instructors are required to use a proactive approach, and they should try to provide pleasant contexts for learners through integrating enjoyment. Moreover, they should reduce apprehension, boredom, and anxiety sources among learners. They should offer instructional materials to increase learners’ enjoyment and pride, boost learners’ cognitive resources, and consolidate learning materials in their minds. Moreover, selecting appropriate materials can lessen negative emotions, such as apprehension and boredom, reduce learners’ cognitive load, and arouse their attentiveness in language learning contexts (Piniel and Albert, 2018). This review implies that instructors can change their engagement and enjoyment both by using different approaches and controlling their outward feelings.

Therefore, L2 instructors require courses to enhance their positive attitudes, including enjoyment and pride, and alleviate negative feelings, such as communication apprehension, boredom, shame, frustration, etc. in their classes. Teachers should express their positive and negative emotions to supervisors and their colleagues. They should utter their challenges and concerns about instructional and organizational problems and educational contexts. Besides, instructive supervisors, who monitor instructors and assess their engagement, can exploit the related studies by considering the instructors’ interpersonal and intrapersonal behaviors. Lack of regulating or controlling teachers’ affections may diminish the enjoyment of both learners and teachers, which may trigger teacher educators to consider this issue in practical aspects. They can provide regulatory strategies for teachers by training them to change their attitudes, raise their awareness about the internal and external sources of boredom, apprehension, and frustration, and develop their objectives along with their individualized achievement. This review recommends that teacher educators should have a positive view toward teachers and learners, and they should provide well organized and inspiring teaching methodologies which can construct a positive context for language learning and teaching, and increase teachers’ wellbeing, excitement, and enjoyment to engage in the classroom. This review can also inspire school principals and educational policymakers to brood over EFL teachers’ traits and their negative and positive emotions. Policymakers should incorporate features of their socio-emotional abilities into their instructive strategies. They are required to organize and design curricula that reduce teacher boredom, apprehension, frustration, and shame, and increase their enjoyment, pride, and work engagement. They can hold intervention programs and workshops to consider both positive and negative feelings. For instance, one of the sources of teachers’ shame and apprehension is their incapability to respond learners’ questions. Teacher educators and administrators can provide some coping strategies for coping with these conditions to lessen these negative emotions. According to Chang (2009, p. 12) “strategies, such as task-focused coping, emotion-focused coping, regaining-task-focused processing, importance reappraisal, and proactive coping are helpful for teachers.”. Moreover, mentors should develop teachers’ confidence in education by providing some exercises, such as presenting materials in class or interviewing with them frequently. The schools and institutes’ managers should provide EFL contexts to support teachers’ work engagement and enjoyment by offering authenticate, joyful, and updated materials to teachers and learners.

This review has some suggestions for further research. Future studies may consist of investigating the influence of other teacher variables, including extroversion and introversion. The causes of positive and negative emotions should be investigated. Future studies can also validate numerous measures of teachers’ apprehension, boredom, frustration, enjoyment, and pride. Investigations need to be done to study teachers’ positive and negative emotions in numerous instructive, local, national, and cultural contexts. Studies should be done on the relationships between teachers’ positive and negative emotions, and their professional progress. Studies can also measure the effect teachers’ emotional regulation on their achievement and job performance.

More studies need to be done on the effect of instructors’ methodologies on their enjoyment and pride. Also, future studies can highlight gender’s effect on language teachers’ enjoyment and pride. Besides, further research needs to be done on the effects of teachers’ positive emotions on their working memory. Likewise, the effect of EFL teachers’ positive emotions on their language proficiency and skills should be meticulously considered. Similarly, the relationships between teachers’ positive and negative feelings are worth to be considered.

## Data Availability Statement

The original contributions presented in the study are included in the article/Supplementary Material, and further inquiries can be directed to the corresponding author.

## Author Contributions

The author confirms being the sole contributor of this work and has approved it for publication.

## Funding

This study was sponsored by the Social Science Planning and Research Project of Shandong Province, “Research on the Construction and Practice of College English Virtual Teaching Environment in the Era of Artificial Intelligence” (grant no. 20CWZj16).

## Conflict of Interest

The author declares that the research was conducted in the absence of any commercial or financial relationships that could be construed as a potential conflict of interest.

## Publisher’s Note

All claims expressed in this article are solely those of the authors and do not necessarily represent those of their affiliated organizations, or those of the publisher, the editors and the reviewers. Any product that may be evaluated in this article, or claim that may be made by its manufacturer, is not guaranteed or endorsed by the publisher.
